# Guanidinium 3-carb­oxy-2,3-dihydroxy­propanoate monohydrate

**DOI:** 10.1107/S1600536809037313

**Published:** 2009-09-19

**Authors:** Mohammad T. M. Al-Dajani, Hassan H. Abdallah, Nornisah Mohamed, Jia Hao Goh, Hoong-Kun Fun

**Affiliations:** a School of Pharmaceutical Sciences, Universiti Sains Malaysia, 11800 USM, Penang, Malaysia; b School of Chemical Sciences, Universiti Sains Malaysia, 11800 USM, Penang, Malaysia; c X-ray Crystallography Unit, School of Physics, Universiti Sains Malaysia, 11800 USM, Penang, Malaysia

## Abstract

In the title hydrated salt, CH_6_N_3_^+^·C_4_H_5_O_6_^−^·H_2_O, the deprotonated carboxyl group is disordered over two positions with a site-occupancy ratio of 0.945 (3):0.055 (3). The bond lengths in the guanidinium cation are inter­mediate between normal C—N and C=N bond lengths, indicating significant delocalization in this species. In the crystal structure, anions and water mol­ecules are linked into sheets parallel to the *ab* plane by inter­molecular O—H⋯O hydrogen bonds. The linking of the anions and water mol­ecules with the cations by inter­molecular N—H⋯O hydrogen bonds creates a three-dimensional network.

## Related literature

For general background to and applications of guanidine derivatives, see: Angyal & Warburton (1951 ▶); Raczyńska *et al.* (2003 ▶); Yamada *et al.* (2009 ▶). For closely related guanidinium structures, see: Najafpour *et al.* (2007 ▶); Pereira Silva *et al.* (2007 ▶). For bond-length data, see: Allen *et al.* (1987 ▶). For the stability of the temperature controller used for the data collection, see: Cosier & Glazer (1986 ▶).
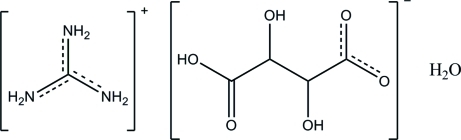


## Experimental

### 

#### Crystal data

CH_6_N_3_^+^·C_4_H_5_O_6_^−^·H_2_O
*M* *_r_* = 227.18
Triclinic, 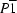

*a* = 7.4588 (1) Å
*b* = 8.0931 (1) Å
*c* = 8.6423 (1) Å
α = 72.415 (1)°
β = 71.620 (1)°
γ = 81.558 (1)°
*V* = 471.18 (1) Å^3^
*Z* = 2
Mo *K*α radiation
μ = 0.15 mm^−1^
*T* = 100 K
0.45 × 0.32 × 0.14 mm

#### Data collection

Bruker SMART APEXII CCD area-detector diffractometer
Absorption correction: multi-scan (**SADABS**; Bruker, 2005 ▶) *T*_min_ = 0.937, *T*_max_ = 0.979
10837 measured reflections
3418 independent reflections
3115 reflections with *I* > 2σ(*I*)
*R*_int_ = 0.018

#### Refinement

*R*[*F* ^2^ > 2σ(*F* ^2^)] = 0.034
*wR*(*F* ^2^) = 0.093
*S* = 1.02
3418 reflections
197 parameters
H atoms treated by a mixture of independent and constrained refinement
Δρ_max_ = 0.49 e Å^−3^
Δρ_min_ = −0.27 e Å^−3^

### 

Data collection: *APEX2* (Bruker, 2005 ▶); cell refinement: *SAINT* (Bruker, 2005 ▶); data reduction: *SAINT*; program(s) used to solve structure: *SHELXTL* (Sheldrick, 2008 ▶); program(s) used to refine structure: *SHELXTL*; molecular graphics: *SHELXTL*; software used to prepare material for publication: *SHELXTL* and *PLATON* (Spek, 2009 ▶).

## Supplementary Material

Crystal structure: contains datablocks global, I. DOI: 10.1107/S1600536809037313/tk2542sup1.cif

Structure factors: contains datablocks I. DOI: 10.1107/S1600536809037313/tk2542Isup2.hkl

Additional supplementary materials:  crystallographic information; 3D view; checkCIF report

## Figures and Tables

**Table 1:** Hydrogen-bond geometry (Å, °)

*D*—H⋯*A*	*D*—H	H⋯*A*	*D*⋯*A*	*D*—H⋯*A*
O2—H1*O*2⋯O5^i^	0.82	1.72	2.5272 (10)	170
O3—H1*O*3⋯O6^ii^	0.836 (16)	1.832 (16)	2.6564 (9)	168.6 (16)
O4—H1*O*4⋯O1*W*^iii^	0.852 (16)	1.963 (16)	2.7455 (10)	152.1 (15)
N1—H1*N*1⋯O1*W*^iv^	0.845 (16)	2.184 (16)	3.0019 (11)	162.8 (15)
N1—H2*N*1⋯O6^iv^	0.859 (16)	2.075 (16)	2.8573 (11)	151.2 (14)
N2—H1*N*2⋯O1	0.844 (16)	2.274 (16)	3.0131 (10)	146.3 (15)
N2—H2*N*2⋯O4^v^	0.854 (15)	2.036 (15)	2.8828 (10)	170.7 (15)
N3—H1*N*3⋯O5^vi^	0.862 (16)	2.049 (16)	2.8973 (11)	167.6 (15)
N3—H2*N*3⋯O1	0.847 (16)	2.441 (16)	3.1540 (11)	142.3 (14)
N3—H2*N*3⋯O3	0.847 (16)	2.345 (16)	3.0410 (11)	139.7 (14)
O1*W*—H1*W*1⋯O3^ii^	0.82 (2)	2.14 (2)	2.9051 (10)	155.0 (15)
O1*W*—H2*W*1⋯O1^vii^	0.842 (16)	1.984 (16)	2.8100 (11)	166.4 (16)

Symmetry codes: (i) 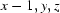; (ii) 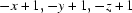; (iii) 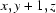; (iv) 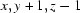; (v) 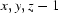; (vi) 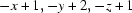; (vii) 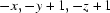.

**Fig. 1.**
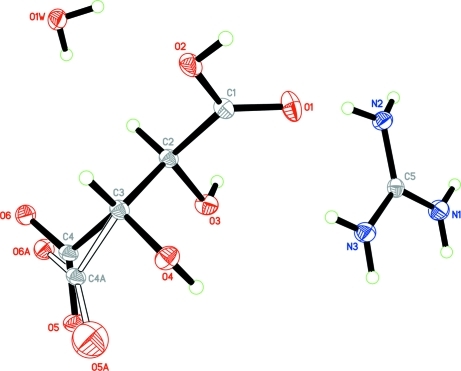
The molecular structure of (I), showing 50% probability displacement ellipsoids for non-H atoms and the atom-numbering scheme. Open bonds indicate the minor disordered component.

**Fig. 2.**
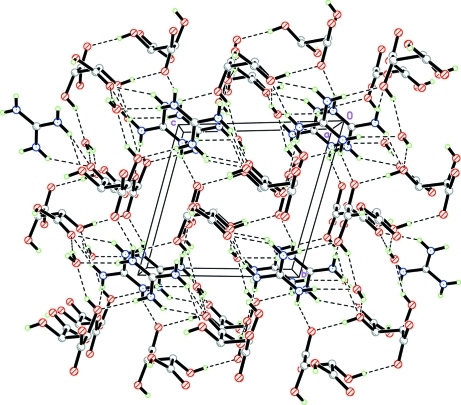
Unit cell contents of (I) viewed along the a axis, showing the three-dimensional network. Only the major component of the anion is shown. H atoms not involved in intermolecular interactions (dashed lines) have been omitted for clarity.

## supplementary crystallographic information

### Comment

Guanidine, formed by the oxidation of guanine, is a strongly alkaline compound that can be used in the manufacturing of plastics and explosives. It is also the final product of the protein metabolism. Interest in this molecule spans many generations of chemists (Angyal & Warburton, 1951; Raczyńska *et al.*, 2003; Yamada *et al.*, 2009).

The asymmetric unit of the title salt (Fig. 1) contains a guanidinium cation, a 3-carboxy-2,3-dihydroxypropanoate anion and a water molecule. A proton transfer from the carboxyl group of 3-carboxy-2,3-dihydroxypropanoic acid to atom N1 of guanidine resulted in the formation of ions. The deprotonated carboxyl group is disordered over two positions with a site-occupancy ratio of 0.945 (3):0.055 (3). The C5—N1, C5—N2 and C5—N3 bond lengths in the propeller-shaped guanidinium cation (CN_3_H_6_)^+^ are almost equal [range of C—N = 1.3286 (10) – 1.3355 (10) Å], indicating that the usual model of electron dislocalization in this species (Allen *et al.*, 1987). The bond lengths and angles are comparable to those found in closely related structures (Najafpour *et al.*, 2007; Pereira Silva *et al.*, 2007).

The crystal structure is mainly stabilized by a network of O—H···O and N—H···O hydrogen bonds. In this network, the O atoms of anion and water molecule act as donors as well as acceptors. Each guanidinium-H atom participates in intermolecular hydrogen bonds. In the crystal structure (Fig. 2), the anions and water molecules are linked into sheets parallel to the *ab* plane by intermolecular O2—H1O2···O5, O3—H1O3···O6, O4—H1O4···O1W, O1W—H1W1···O3 and O1W—H2W1···O1 hydrogen bonds (Table 1). The anions and water molecules are further linked with the cations by intermolecular N1—H1N1···O1W, N1—H2N1···O6, N2—H1N2···O1, N2—H2N2···O4, N3—H1N3···O5, N3—H2N3···O1 and N3—H2N3···O3 hydrogen bonds (Table 1), thus establishing a connection between these sheets to create a three-dimensional crystal structure.

### Experimental

Tartaric acid (1 mol) was dissolved in THF (10 ml) in a round bottom flask. In a separating funnel, guanidine carbonate (1 mol), 99 % [H_2_NC(=NH)NH_2_].2H_2_CO_3_, was dissolved in THF (10 ml) and three drops of concentrated HCl were added. The guanidine solution then was added drop-wise to the flask of tartaric acid with stirring. The reactant mixture was left stirring for 3 h at room temperature. The colourless single crystals formed were washed with THF and dried at 353 K.

### Refinement

Atom H1O2 was placed in a calculated position, with O—H = 0.82 Å and *U*_iso_ = 1.5*U*_eq_(O), and was refined using a freely rotating O—H bond. The other H atoms were located from difference Fourier map and allowed to refine freely, range of C—H = 0.945 (13)–1.015 (13) Å. The carboxylate group is disordered over two positions with a site-occupancy ratio of 0.945 (3):0.055 (3). For the minor disordered component, only the C atom was refined anisotropically.

### Crystal data

**Table:**

CH_6_N_3_^+^·C_4_H_5_O_6_^−^·H_2_O	*Z* = 2
*M**_r_* = 227.18	*F*(000) = 240
Triclinic, *P*1	*D*_x_ = 1.601 Mg m^−^^3^
Hall symbol: -P 1	Mo *K*α radiation, λ = 0.71073 Å
*a* = 7.4588 (1) Å	Cell parameters from 6392 reflections
*b* = 8.0931 (1) Å	θ = 2.6–32.6°
*c* = 8.6423 (1) Å	µ = 0.15 mm^−^^1^
α = 72.415 (1)°	*T* = 100 K
β = 71.620 (1)°	Block, colourless
γ = 81.558 (1)°	0.45 × 0.32 × 0.14 mm
*V* = 471.18 (1) Å^3^	

### Data collection

**Table:**

Bruker SMART APEXII CCD area-detector diffractometer	3418 independent reflections
Radiation source: fine-focus sealed tube	3115 reflections with *I* > 2σ(*I*)
graphite	*R*_int_ = 0.018
φ and ω scans	θ_max_ = 32.6°, θ_min_ = 2.6°
Absorption correction: multi-scan (*SADABS*; Bruker, 2005)	*h* = −11→11
*T*_min_ = 0.937, *T*_max_ = 0.979	*k* = −11→12
10837 measured reflections	*l* = −12→13

### Refinement

**Table:**

Refinement on *F*^2^	Primary atom site location: structure-invariant direct methods
Least-squares matrix: full	Secondary atom site location: difference Fourier map
*R*[*F*^2^ > 2σ(*F*^2^)] = 0.034	Hydrogen site location: inferred from neighbouring sites
*wR*(*F*^2^) = 0.093	H atoms treated by a mixture of independent and constrained refinement
*S* = 1.02	*w* = 1/[σ^2^(*F*_o_^2^) + (0.0471*P*)^2^ + 0.1755*P*] where *P* = (*F*_o_^2^ + 2*F*_c_^2^)/3
3418 reflections	(Δ/σ)_max_ < 0.001
197 parameters	Δρ_max_ = 0.49 e Å^−^^3^
0 restraints	Δρ_min_ = −0.27 e Å^−^^3^

### Special details

**Table:**

Experimental. The crystal was placed in the cold stream of an Oxford Cyrosystems Cobra open-flow nitrogen cryostat (Cosier & Glazer, 1986) operating at 100.0 (1)K.
Geometry. All esds (except the esd in the dihedral angle between two l.s. planes) are estimated using the full covariance matrix. The cell esds are taken into account individually in the estimation of esds in distances, angles and torsion angles; correlations between esds in cell parameters are only used when they are defined by crystal symmetry. An approximate (isotropic) treatment of cell esds is used for estimating esds involving l.s. planes.
Refinement. Refinement of F^2^ against ALL reflections. The weighted R-factor wR and goodness of fit S are based on F^2^, conventional R-factors R are based on F, with F set to zero for negative F^2^. The threshold expression of F^2^ > 2sigma(F^2^) is used only for calculating R-factors(gt) etc. and is not relevant to the choice of reflections for refinement. R-factors based on F^2^ are statistically about twice as large as those based on F, and R- factors based on ALL data will be even larger.

### Fractional atomic coordinates and isotropic or equivalent isotropic displacement parameters (Å^2^)

**Table:**

	*x*	*y*	*z*	*U*_iso_*/*U*_eq_	Occ. (<1)
O1	−0.00986 (9)	0.73134 (10)	0.50517 (8)	0.02036 (14)	
O2	−0.09291 (9)	0.54671 (9)	0.76558 (8)	0.01726 (13)	
H1O2	−0.2005	0.5811	0.7604	0.026*	
O3	0.35923 (9)	0.69520 (8)	0.49515 (8)	0.01562 (12)	
O4	0.19600 (9)	0.77452 (8)	0.81295 (8)	0.01573 (12)	
C1	0.02941 (11)	0.62976 (11)	0.62883 (10)	0.01333 (14)	
C2	0.23376 (10)	0.58428 (10)	0.63385 (9)	0.01179 (13)	
C3	0.25872 (11)	0.60619 (10)	0.79571 (9)	0.01189 (13)	
C4	0.46665 (12)	0.55432 (14)	0.78966 (10)	0.01126 (17)	0.945 (3)
O5	0.57222 (9)	0.66863 (9)	0.77930 (9)	0.01673 (17)	0.945 (3)
O6	0.52211 (9)	0.39998 (9)	0.79084 (8)	0.01420 (16)	0.945 (3)
C4A	0.480 (3)	0.606 (3)	0.793 (2)	0.01126 (17)	0.055 (3)
O5A	0.503 (3)	0.736 (3)	0.823 (3)	0.042 (5)*	0.055 (3)
O6A	0.5654 (17)	0.4820 (18)	0.7605 (14)	0.015 (3)*	0.055 (3)
N1	0.29076 (11)	1.10920 (10)	−0.04338 (10)	0.01787 (14)	
N2	0.13002 (11)	0.86542 (10)	0.12497 (9)	0.01554 (13)	
N3	0.25830 (11)	1.02160 (10)	0.24365 (10)	0.01706 (14)	
C5	0.22818 (11)	0.99832 (10)	0.10812 (10)	0.01293 (14)	
O1W	0.31892 (10)	0.09289 (9)	0.60724 (9)	0.01863 (13)	
H2A	0.2663 (17)	0.4572 (16)	0.6371 (16)	0.011 (3)*	
H3A	0.1831 (18)	0.5272 (17)	0.8903 (17)	0.014 (3)*	
H1O3	0.382 (2)	0.660 (2)	0.409 (2)	0.032 (4)*	
H1O4	0.261 (2)	0.851 (2)	0.731 (2)	0.035 (4)*	
H1N1	0.277 (2)	1.093 (2)	−0.131 (2)	0.029 (4)*	
H2N1	0.356 (2)	1.194 (2)	−0.0562 (19)	0.024 (3)*	
H1N2	0.112 (2)	0.789 (2)	0.219 (2)	0.029 (4)*	
H2N2	0.136 (2)	0.8370 (19)	0.0359 (19)	0.023 (3)*	
H1N3	0.325 (2)	1.105 (2)	0.233 (2)	0.026 (3)*	
H2N3	0.227 (2)	0.944 (2)	0.337 (2)	0.025 (3)*	
H1W1	0.417 (3)	0.143 (2)	0.551 (2)	0.039 (4)*	
H2W1	0.240 (2)	0.154 (2)	0.559 (2)	0.036 (4)*	

### Atomic displacement parameters (Å^2^)

**Table:**

	*U*^11^	*U*^22^	*U*^33^	*U*^12^	*U*^13^	*U*^23^
O1	0.0161 (3)	0.0283 (3)	0.0131 (3)	0.0031 (2)	−0.0054 (2)	−0.0013 (2)
O2	0.0102 (2)	0.0224 (3)	0.0168 (3)	−0.0034 (2)	−0.0044 (2)	−0.0001 (2)
O3	0.0144 (3)	0.0197 (3)	0.0112 (2)	−0.0052 (2)	−0.00005 (19)	−0.0039 (2)
O4	0.0180 (3)	0.0138 (3)	0.0148 (3)	−0.0006 (2)	−0.0026 (2)	−0.0054 (2)
C1	0.0121 (3)	0.0160 (3)	0.0126 (3)	−0.0009 (2)	−0.0042 (2)	−0.0042 (3)
C2	0.0100 (3)	0.0140 (3)	0.0113 (3)	−0.0016 (2)	−0.0027 (2)	−0.0032 (2)
C3	0.0101 (3)	0.0138 (3)	0.0116 (3)	−0.0012 (2)	−0.0029 (2)	−0.0030 (2)
C4	0.0101 (3)	0.0143 (4)	0.0099 (3)	−0.0040 (3)	−0.0026 (2)	−0.0027 (3)
O5	0.0117 (3)	0.0165 (3)	0.0236 (3)	−0.0037 (2)	−0.0053 (2)	−0.0063 (2)
O6	0.0133 (3)	0.0135 (3)	0.0157 (3)	−0.0003 (2)	−0.0044 (2)	−0.0040 (2)
C4A	0.0101 (3)	0.0143 (4)	0.0099 (3)	−0.0040 (3)	−0.0026 (2)	−0.0027 (3)
N1	0.0182 (3)	0.0184 (3)	0.0147 (3)	−0.0050 (3)	−0.0037 (2)	−0.0003 (3)
N2	0.0188 (3)	0.0143 (3)	0.0139 (3)	−0.0032 (2)	−0.0050 (2)	−0.0030 (2)
N3	0.0212 (3)	0.0164 (3)	0.0156 (3)	−0.0027 (3)	−0.0073 (3)	−0.0043 (3)
C5	0.0112 (3)	0.0133 (3)	0.0136 (3)	0.0011 (2)	−0.0034 (2)	−0.0036 (2)
O1W	0.0148 (3)	0.0186 (3)	0.0192 (3)	−0.0026 (2)	−0.0058 (2)	0.0013 (2)

### Geometric parameters (Å, °)

**Table:**

O1—C1	1.2246 (10)	C4—O5	1.2662 (12)
O2—C1	1.3042 (10)	C4A—O6A	1.17 (2)
O2—H1O2	0.8200	C4A—O5A	1.20 (3)
O3—C2	1.4169 (9)	N1—C5	1.3286 (10)
O3—H1O3	0.840 (18)	N1—H1N1	0.844 (17)
O4—C3	1.4101 (10)	N1—H2N1	0.859 (15)
O4—H1O4	0.851 (18)	N2—C5	1.3355 (10)
C1—C2	1.5258 (11)	N2—H1N2	0.846 (16)
C2—C3	1.5319 (11)	N2—H2N2	0.854 (16)
C2—H2A	1.015 (13)	N3—C5	1.3303 (10)
C3—C4	1.5347 (11)	N3—H1N3	0.865 (16)
C3—C4A	1.641 (18)	N3—H2N3	0.851 (15)
C3—H3A	0.945 (13)	O1W—H1W1	0.822 (19)
C4—O6	1.2549 (12)	O1W—H2W1	0.842 (18)
			
C1—O2—H1O2	109.5	C4A—C3—H3A	115.5 (10)
C2—O3—H1O3	109.6 (11)	O6—C4—O5	124.13 (8)
C3—O4—H1O4	110.8 (12)	O6—C4—C3	116.93 (8)
O1—C1—O2	125.21 (8)	O5—C4—C3	118.92 (8)
O1—C1—C2	121.76 (7)	O6A—C4A—O5A	140 (2)
O2—C1—C2	113.01 (7)	O6A—C4A—C3	110.4 (14)
O3—C2—C1	111.04 (6)	O5A—C4A—C3	109.7 (16)
O3—C2—C3	107.06 (6)	C5—N1—H1N1	121.2 (11)
C1—C2—C3	110.81 (6)	C5—N1—H2N1	120.5 (10)
O3—C2—H2A	112.1 (7)	H1N1—N1—H2N1	118.0 (14)
C1—C2—H2A	109.1 (7)	C5—N2—H1N2	115.7 (11)
C3—C2—H2A	106.6 (7)	C5—N2—H2N2	118.2 (10)
O4—C3—C2	111.00 (6)	H1N2—N2—H2N2	119.5 (14)
O4—C3—C4	115.34 (7)	C5—N3—H1N3	120.5 (10)
C2—C3—C4	106.58 (6)	C5—N3—H2N3	118.7 (10)
O4—C3—C4A	99.2 (7)	H1N3—N3—H2N3	119.9 (15)
C2—C3—C4A	114.6 (6)	N1—C5—N3	120.41 (8)
C4—C3—C4A	16.1 (7)	N1—C5—N2	119.70 (7)
O4—C3—H3A	107.2 (8)	N3—C5—N2	119.87 (7)
C2—C3—H3A	108.8 (8)	H1W1—O1W—H2W1	101.2 (17)
C4—C3—H3A	107.8 (8)		
			
O1—C1—C2—O3	10.22 (11)	C2—C3—C4—O6	−62.98 (9)
O2—C1—C2—O3	−171.38 (7)	C4A—C3—C4—O6	175 (2)
O1—C1—C2—C3	129.07 (8)	O4—C3—C4—O5	−8.52 (11)
O2—C1—C2—C3	−52.53 (9)	C2—C3—C4—O5	115.16 (8)
O3—C2—C3—O4	66.54 (8)	C4A—C3—C4—O5	−7(2)
C1—C2—C3—O4	−54.69 (8)	O4—C3—C4A—O6A	−172.1 (12)
O3—C2—C3—C4	−59.80 (8)	C2—C3—C4A—O6A	−53.8 (14)
C1—C2—C3—C4	178.98 (7)	C4—C3—C4A—O6A	9.2 (12)
O3—C2—C3—C4A	−44.8 (7)	O4—C3—C4A—O5A	6.8 (16)
C1—C2—C3—C4A	−166.1 (7)	C2—C3—C4A—O5A	125.0 (14)
O4—C3—C4—O6	173.34 (7)	C4—C3—C4A—O5A	−172 (3)

### Hydrogen-bond geometry (Å, °)

**Table:**

*D*—H···*A*	*D*—H	H···*A*	*D*···*A*	*D*—H···*A*
O2—H1O2···O5^i^	0.82	1.72	2.5272 (10)	170
O3—H1O3···O6^ii^	0.836 (16)	1.832 (16)	2.6564 (9)	168.6 (16)
O4—H1O4···O1W^iii^	0.852 (16)	1.963 (16)	2.7455 (10)	152.1 (15)
N1—H1N1···O1W^iv^	0.845 (16)	2.184 (16)	3.0019 (11)	162.8 (15)
N1—H2N1···O6^iv^	0.859 (16)	2.075 (16)	2.8573 (11)	151.2 (14)
N2—H1N2···O1	0.844 (16)	2.274 (16)	3.0131 (10)	146.3 (15)
N2—H2N2···O4^v^	0.854 (15)	2.036 (15)	2.8828 (10)	170.7 (15)
N3—H1N3···O5^vi^	0.862 (16)	2.049 (16)	2.8973 (11)	167.6 (15)
N3—H2N3···O1	0.847 (16)	2.441 (16)	3.1540 (11)	142.3 (14)
N3—H2N3···O3	0.847 (16)	2.345 (16)	3.0410 (11)	139.7 (14)
O1W—H1W1···O3^ii^	0.82 (2)	2.14 (2)	2.9051 (10)	155.0 (15)
O1W—H2W1···O1^vii^	0.842 (16)	1.984 (16)	2.8100 (11)	166.4 (16)

Symmetry codes: (i) *x*−1, *y*, *z*; (ii) −*x*+1, −*y*+1, −*z*+1; (iii) *x*, *y*+1, *z*; (iv) *x*, *y*+1, *z*−1; (v) *x*, *y*, *z*−1; (vi) −*x*+1, −*y*+2, −*z*+1; (vii) −*x*, −*y*+1, −*z*+1.
